# Capacity for One Health research in the Horn of Africa

**DOI:** 10.1016/j.onehlt.2023.100549

**Published:** 2023-04-28

**Authors:** K. Marie McIntyre, Michael Cooper, Matthew Baylis

**Affiliations:** a Department of Livestock and One Health, Institute of Infection, Veterinary and Ecological Sciences, University of Liverpool, IC2 Building, Brownlow Hill, Liverpool L3 5RF, United Kingdom; b Network for EcoHealth and One Health (NEOH), European Chapter of Ecohealth International, Kreuzstrasse 2, P.O. Box, 4123 Allschwil, Switzerland

**Keywords:** One Health, Horn of Africa, Human-animal-environment dimensions, Research funding, Research bias, Laboratory, Environment neglect

## Abstract

**Introduction:**

In low-and-middle-income countries, many people live near livestock. Rural livelihoods need improvement, however livestock-sector growth is a ‘wicked’ problem, needing careful management and One Health approaches which balance positive aspects of livestock ownership against deleterious impacts.

**Materials and methods:**

A Key Informant survey was delivered to higher education and research institute Units in Horn of Africa, to quantify baseline estimates for One Health research, understand characteristics, and risk factors for usage.

**Principal results:**

Four-fifths of Units acknowledged some One Health research; however, this was biased towards human-focused dimensions including at the human/animal/environment-interface and human/animal-interface; One Health approaches were also more often reported when all or the animal/environment dimensions were examined. We detected subject-bias impacting environment-focus in research; only research-focused Units had staff with higher environmental science degrees. Our work suggested good national research buy-in, and Units engaging with national policy-makers most often; local policy-makers were least engaged. Four-fifths of Units had laboratories, with two-thirds processing either human or animal samples and half processing both. Funding for equipment purchase, supplies and maintenance, staff training on technical/safety issues was nearly half that previously identified.

**Major conclusions:**

The necessity for One Health research approaches is acknowledged, however our results suggest persistent and systemic neglect of the environment in approaches and research staff education, and a lack of integration across government hierarchies during policy-development, potentially driven by international organisation domination. Further, Units lack funding for laboratory equipment purchase/supplies/maintenance, and staff training on technical/safety issues.

## 1 Introduction

The COVID-19 pandemic exemplified the need to consider using One Health (OH) approaches to efficiently resolve some health challenges [1]. Substantial and persistent inequalities in health spending and service delivery have been exacerbated by the pandemic, moving a global Universal Health Coverage target further away [2]. Almost unanimously across (European) countries, in lower socioeconomic groups, poorer health self-assessments and death rates are linked [3]. In low- and middle-income countries e.g., in Horn of Africa, many people live close to livestock, which provide food and income. It is therefore vital to improve rural population livelihoods; livestock-sector growth, however, is a ‘wicked’ problem, needing careful monitoring and evaluation of management practices and a OH lens to balance positive livestock ownership aspects against deleterious impacts in circumstance-specific scenarios. These include increased zoonotic and foodborne disease transmission risks with livestock ownership, and environmental impacts such as land degradation resulting from overgrazing, emissions of greenhouse gases and water pollution due to manure disposal [4]. In fragile ecosystems such as the Horn of Africa, livestock-sector development sustains rural livelihoods; it is essential to understand current research perspectives, recognising available research infrastructures. Once clarified, solid development plans and capacity-building actions will ensure that OH research is best developed to ameliorate health issues.

The HORN project (One Health Regional Network for the Horn of Africa) intended to better understand research capabilities in the Horn of Africa, propose plans and implement these to strengthen organisational abilities to undertake OH research. For this, an online survey was delivered, aiming to quantify baseline estimates for OH research in individual departments within higher education (HE) and research institute (RI) organisations, using a Key Informant approach. The survey also sought to understand the characteristics and risk factors for OH approach usage, identifying strengths, weaknesses and gaps in regional OH working, to focus capacity-building objectives. The work followed previous studies; a systematic analysis of OH networks delivered to understand duplication of efforts in geographical scope, human/animal/environmental sectors, activities and stakeholder engagement [5], and characterisation of institutional facilities and supporting research infrastructure in national health research systems [6].

## 2 Materials and methods

### 2.1 Questionnaire design and administration

A Key Informant survey was delivered to HE and RIs in Eritrea, Ethiopia, Kenya, Somalia and Somaliland using an online questionnaire. Key Informants were senior staff members e.g., administrative officials, department heads and research directors for each department (or College) in HEs/RIs. They were identified in advance by: prior contact with the HORN project; undertaking searches using the internet; or examining author affiliations in OH research-focused peer-reviewed publications and grey literature (see Appendix A). To avoid replication of responses for HEs/RIs, answers provided by the most senior responding staff member were used.

Survey questions focused on Unit characteristics, research disciplines and topics, dissemination capabilities, access to OH networks and laboratory facilities. Research “Units” are described in analyses, tailored to target institutes e.g., most senior staff member in university/RI College/department, or standalone RI, if smaller departments were inappropriate.

### 2.2 Statistical analyses

Data was examined using MS Excel, R [7], ggplot2 [8] and ggtern [9] graphics packages, with statistical significance determined by alpha level = 0.050. Two-sided Fisher's Exact or Chi-square tests were used to examine associations between categorical data, with *P*-values computed using Monte Carlo simulation, if necessary. After rejecting null hypotheses, post-hoc pairwise testing used the *fisher.multcomp* function from *RVAideMemoire*package [10] to examine differences between groups. Patterns in OH dimensions of Unit topics were examined using binary logistic regression modelling with a logit link-function, with counts of Units described by halving the distribution.

## 3 Results

The Survey Invitation was sent to 1833 potential respondents. In total, *N* = 228 consented to its completion, a response rate of 15.7% (assuming no invitation cascading). Of these, *N* = 159 responses were used in analyses, after replicate Unit responses removal.

### 3.1 Research focus and funding of Units

**Fig. 1:**
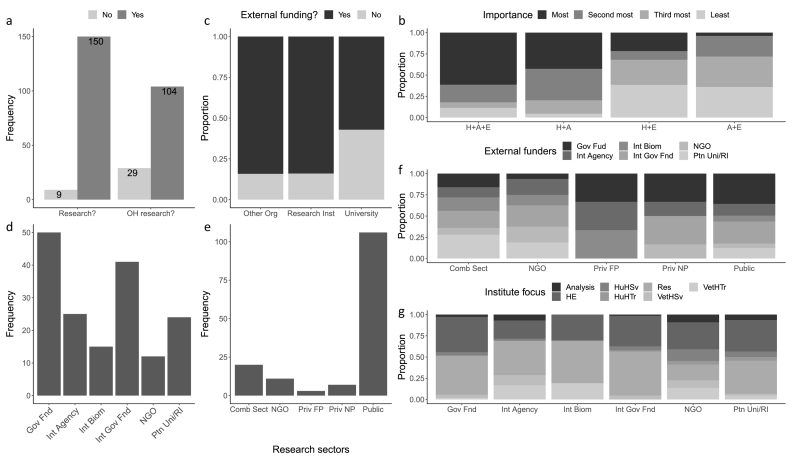
a. Frequency of research Units reporting undertaking research (Research?) and research in which a One Health approach is used (OH research?). Data labels are frequency of research Units reporting. Fig. 1b. Proportion of research mostly like to be focused on humans, animals and the environment (H ± A ± E), humans and animals (H ± A), humans and the environment (H ± E), and animals and the environment (A ± E) (ranked by level of importance) by research Unit. Fig. 1c. Proportion of research Units reporting receiving external funding by type of institute including Research Institute (Research Inst), University, and Other Organisations (Other Org). Fig. 1d. Frequency of research Units reporting different types of external organisations as their main funders. Fig. 1e. Frequency of research Units belonging to certain sectors. Fig. 1f. Proportion of research Units reporting receiving funding from their main external funders by research sector and Fig. 1g. primary function of their institution. *External funders* include: national government funding (Gov Fnd), international agencies (Int Agency, e.g., FAO, WHO, World Bank), international biomedical research charities (Int Biom, e.g., Wellcome Trust), international government funding organisations (Int Gov Fnd, e.g., USAID, NoRAD, JICA, DFG), NGOs, and partner universities/ research institutes (Ptn Uni/RI). No response to external funders was provided by N = 15 research Units, and *N* = 23 responded that they do not receive external funding. *Research sectors* include: combination of different sectors (Comb Sect), NGOs, private for-profit (Priv FP), private not-for-profit (Priv NP) and public (Public). One research Unit was described as being within the ‘Clinical research’ sector, and no response to research sector was provided by *N* = 1. *Primary functions* include providing analysis and/or supporting decision-making in government or civil services (Analysis), being Higher Education establishments (HE), providing human health services (HuHSv), providing human health training (HuHTr), providing veterinary health services (VetHSv), providing veterinary health training (VetHTr) and conducting research (Res). Note: Research Units could provide two main external funders and two primary functions.

Most (94.3%) respondents reported undertaking research (9 reported no research, so were not considered further) and 78.2% (104/133, no answer = 17) reported some research in Units using OH approaches (Fig. 1a). OH research was most frequently focused on humans, animals and the environment, followed by humans and animals (not environment), then humans and the environment (not animals), and finally animals and the environment (not humans) (Fig. 1b). Units most often had national (*N* = 91, 60.7%) followed by international (*N* = 30, 20.0%), regional (*N* = 16, 10.7%) or local (*N* = 13, 8.7%) focus.

External research funding was received by 64.7% of Units (97/150). RIs and other organisations were 50% (RR = 1.48 and 1.49, respectively) more likely to receive external funding than universities (*P* = 0.035 and *P* = 0.035, respectively; Fig. 1c). Main external Unit funders included national then international government organisations; other funders were less often reported (Fig. 1d). Most Units were in the public sector, followed by a combination of sectors, then NGOs, private for-profit, and not-for-profit (Fig. 1e). Government and international agency funding were important for most research sectors, however for NGO and private sector Units, international biomedical research and government funding were also important (Fig. 1f).

Units had primary functions including conducting research (*N* = 106), providing higher education (*N* = 98), veterinary health training (*N* = 17), human health (*N* = 15) or veterinary health services (*N* = 11), analysis and/or supporting decision-making in government/civil services (*N* = 10), human health training (*N* = 8), and pharmaceutical product development and/or distribution, genomic analysis, information on disease vectors, or community service (*N* = 1 for each). Government, partner university/RIs and international government-funded Units were mostly focused on HE and research compared to international agency or biomedical research and NGO-funded Units which also focused on human and veterinary health services, and training. NGO and international agency-funded Units also had significant analytical focus (Fig. 1g).

### 3.2 Physical and human resource characteristics of Units

**Fig. 2:**
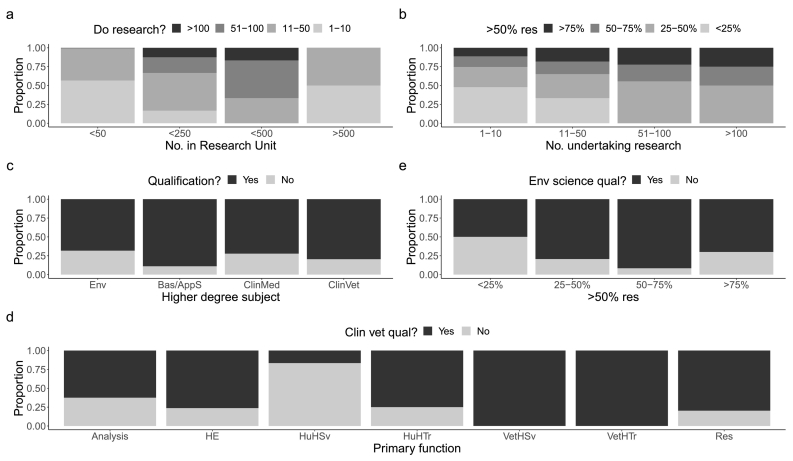
a. Proportion of staff undertaking research in Units by size of research Unit. Fig. 2b. Proportion of staff undertaking research for >50% of their time by number of staff undertaking research in Unit. Fig. 2c. Proportion of research Units in which researchers have higher degree subjects including aligned to environmental sciences (Env), basic or applied sciences (Bas/AppS), clinical medical (ClinMed) or clinical veterinary (ClinVet) topics. Fig. 2d. Proportion of staff with a clinical veterinary qualification by Units' primary function. Note: Research Units could provide two primary functions. Fig. 2e. Proportion of staff with a higher environmental science qualification by the proportion of researchers spending more than half their time doing research. *Primary functions* include providing analysis and/or supporting decision-making in government or civil services (Analysis), being Higher Education establishments (HE), providing human health services (HuHSv), providing human health training (HuHTr), providing veterinary health services (VetHSv), providing veterinary health training (VetHTr) and conducting research (Res).

Units were most often in Ethiopia (*N* = 96, 64.0%), then Kenya (*N* = 38, 25.3%), Somalia and Somaliland (both *N* = 6, 4.0%), in multiple countries (all N = 3, 2.1%), and Eritrea (*N* = 1, 0.7%). The most common official/working language was English (*N* = 121, 80.7%), then Amharic (*N* = 21, 14.0%), Somali (*N* = 7, 4.7%), and Tigrinya (N = 1, 0.7%). Most Units were small, with 113 Units employing <50 people, 24 employing 51–250, 6 employing 251–500, and 6 employing over 500 people (one blank response). Generally, medium-sized Units reported largest numbers of individuals undertaking research (Fig. 2a). In addition, the greater the number undertaking research, the greater the proportion spending more time doing this (Fig. 2b). Most Units employed mostly in-country nationals (>75% of employees were nationals, *N* = 132, 88.0%), with 6.7% (*N* = 10) having 51–75% of employees who were nationals and a smaller number employing lower proportions (26–50% nationals, *N* = 3, 2.0%; and 0–25% nationals, *N* = 5, 3.3%). More than half of Units had very low proportions of women on their staff (0–25%, *N* = 77, 52.7%), with 37.7% (*N* = 55) having good proportions (26–50% women), and low numbers having excellent proportions (51–75% women, *N* = 12, 8.2%, >75% women, *N* = 2, 1.4%, *N* = 4 no response).

Researchers with basic/applied science higher degrees (MSc/MA/PhD) were most often employed in Units, followed by clinical veterinary and medicine, then higher environmental science degrees (MSc/MA/PhD) (Fig. 2c). The likelihood of employing staff with clinical veterinary degrees was substantially increased when Units had certain primary functions compared to those focused on human health services (Fig. 2d): if focused on higher education (RR = 4.58, *P* = 0.032), veterinary health services (RR = 6.00, *P* = 0.021), veterinary health training (RR = 6.00, *P* = 0.005) or undertaking research (RR = 4.79, *P* = 0.021). The likelihood of having researchers with higher degrees in environmental science was increased by >50% when researchers spent >50% of their time undertaking research (Fig. 2e, RR = 1.59 for 25–50% (*P* = 0.048) and RR = 1.83 for 50–75% (*P* = 0.048) compared to for 0–25% of Unit staff).

### 3.3 Laboratory facilities in Units

**Fig. 3:**
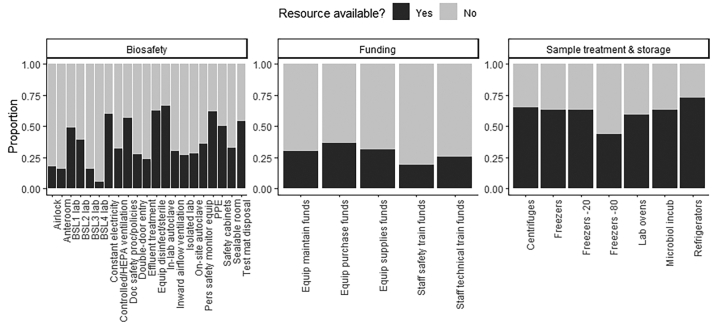
Proportion of research Units in which laboratory resources were reported including biosafety equipment, funding resources to support maintenance, purchase, supplies and training, and sample treatment and storage equipment.

Twenty-nine Units (19.33%) had no laboratory facilities, two-thirds could process human (*N* = 95, 66.9%) or animal samples (N = 95, 67.9%), and over half could process both (*N* = 72, 53.3%). Forty percent (*N* = 54) of Units had National Reference Laboratories, 81.2% (*N* = 117) could send samples to other in-country laboratories, 59.1% (*N* = 81) could send samples to neighbouring-country-based laboratories, and 55.9% (*N* = 76) could send samples to international laboratories. There was variability in laboratory resource availability (Fig. 3) with biosafety resources including laboratory autoclaves most often available (reported for 66.67% of Units), followed by sterilising and disinfection equipment (62.67%), personal protective clothing (62.00%), constant electrical supply (60.00%), and documented safety procedures/policies (56.67%). Lowest availability was for Biosafety Level (BSL)4 (5.33%) and BSL3 (15.33%) sample handling laboratories, anteroom (15.33%) and airlock (17.33%) facilities. Sample treatment and storage facilities were most often available (Fig. 3), including refrigerators (72.67%), centrifuges (65.33%), standard or − 20 °C freezers (63.33% and 63.33%, respectively), microbiological incubators (63.33%), laboratory ovens (59.33%), and least commonly available −80 °C freezers (43.33%). Funding was limited for equipment purchase (36.67%), supplies (31.33%), maintenance (30.00%), and for staff training on technical (25.33%) and safety issues (18.67%).

### 3.4 Characteristics of Units using One Health approaches

**Fig. 4:**
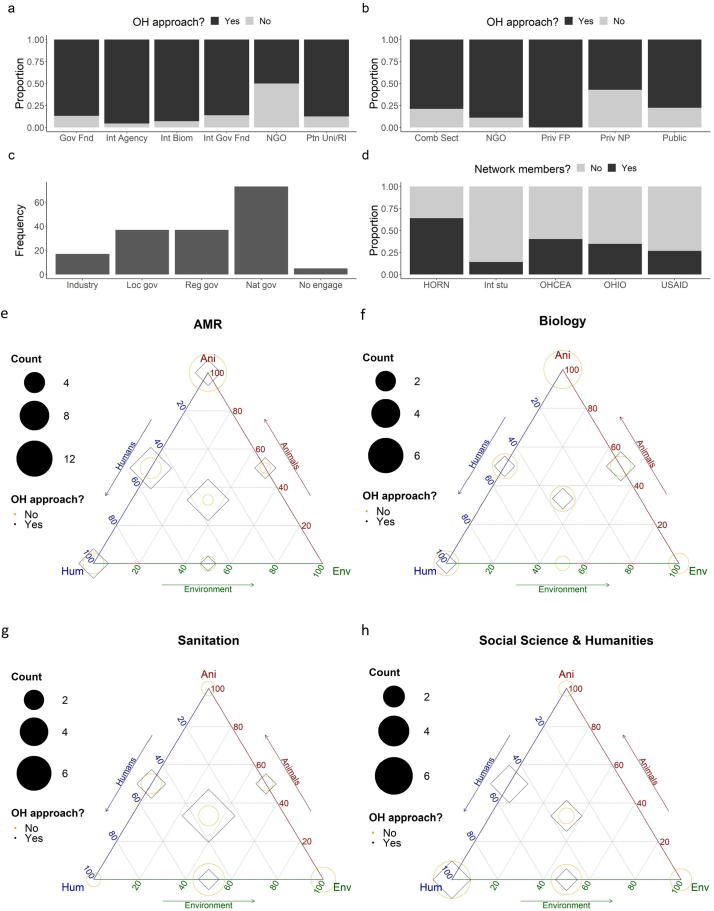
Proportion of research Units undertaking research using a One Health approach, or without such an approach, by their main external research funders (a), or by their by research sector (b). Research sectors include: combination of different sectors (Comb Sect), NGOs, private for-profit (Priv FP), private not-for-profit (Priv NP) and public (Public). Funders include: national government funding (Gov Fnd), international agencies (Int Agency), international biomedical research charities (Int Biom), international government funding organisations (Int Gov Fnd), NGOs, and partner universities/ research institutes (Ptn Uni/RI). Note: Research Units could provide two main external funders. Fig. 4c. Frequency of research Units undertaking research using a One Health ethos that reported engaging with policymakers by type of policymaker. Policymakers include Industry, local (Loc gov), regional (Reg gov) and national (Nat gov) government, and no engagement with policymakers (No engage). Ten respondents either responded they didn't know or left the answer blank. Fig. 4d. Proportion of research Units in which staff were reported as members of One Health networks. Networks include the One Health Regional Network for the Horn of Africa (HORN), the International Student One Health Alliance (Int stu), the Africa One Health University Network/One Health Central and Eastern Africa network (OHCEA), the Ohio State University Global One Health Institute (OHIO) and the One Health workforce project (USAID). Other networks reported include Afrique One (*N* = 1), the National One Health Steering Committee of Ethiopia (*N* = 2), Ecohealth Alliance (N = 2), CRDF Global (N = 1), One Health Commission (N = 1), African One Health Network (N = 1) and the African Science Partnership for Intervention Research Excellence (N = 1). Fig. 4e-h. Patterns in whether research approaches for topics (or disciplines) were more often reported as One Health, and whether the research examined the human, animal and environment dimensions. The counts describe the frequency of Units reporting undertaking research for each topic (or discipline) and the dimensions their research covered. The differences were statistically significant for some comparisons and examples for specific topics are provided by illustration (note, the statistical analysis was comparison of patterns across all topics). A point but no diamond or circle depicts a count of zero; for full results for all topics see Table 1 and Appendix A, Fig. 1. One Health approaches (black diamonds) were more often reported (compared to not using One Health approaches – yellow circles, i.e. black diamonds larger than yellow circles) when the animal, human and environment dimensions were examined for the most popular (top-half) topics compared to when the animal and environment were examined for the least popular (bottom-half) topics (Odds Ratio(OR) = 6.11, Lower Confidence Interval(LCI) = 1.23, Upper Confidence Interval(UCI) = 32.06, *P* > 0.030 - illustrated using antimicrobial resistance (Fig. 4e.) versus biology (Fig. 4f.). One Health approaches were also more often reported compared to not using One Health approaches for the least popular topics examined using the human and environment dimensions (OR = 28.54, LCI = 4.85, UCI = 204.78, *P* > 0.001; illustrated using antimicrobial resistance (Fig. 4e.) versus sanitation (Fig. 4g.). The most popular topics examined using the human and animal dimensions were more often reported to use a One Health approach than the least popular topics examined using the human and environment dimensions (in a borderline relationship - OR = 4.67, LCI = 0.79, UCI = 32.50, *P* = 0.099); illustrated using antimicrobial resistance (Fig. 4e) versus social sciences and humanities (Fig. 4h.). One Health approaches were also more likely to be reported for the most popular topics examined using the animal and environment dimensions compared to the least popular topics examined using the human and environment dimensions (OR = 18.98, LCI = 2.91, UCI = 148.79, *P* > 0.003); illustrated using antimicrobial resistance (Fig. 4e.) versus sanitation (Fig. 4g.).

OH research was more likely to be undertaken if funded by international agencies and biomedical research charities compared to NGOs (borderline differences; *P* = 0.060, Fig. 4a). It was also more likely if research was undertaken in for-profit compared to not-for-profit private Units (not significant differences; *P* = 0.664, Fig. 4b). Staff from most (87.7%, 121/138) Units provided reports to government organisations; three-quarters (77.5%, 100/129) provided reports to NGOs. If undertaking OH research, Unit staff were most likely to engage with national, then regional and local government, and industry policymakers; few had no policymaker engagement (Fig. 4c). There were borderline associations between having laboratory facilities to process animal, or animal and human samples, and undertaking OH research (X^2^ = 3.77, *P* = 0.052 and X^2^ = 2.98, *P* = 0.084, respectively). Unit staff were more likely to be members of certain OH networks e.g., HORN (all pairwise comparisons *P* < 0.001), compared to other networks (Fig. 4d); they were also more likely to be members of Africa One Health University Network (AFROHUN)/One Health Central and Eastern Africa network (OHCEA) or Ohio State University Global One Health Institute compared to International Student One Health Alliance (*P* < 0.001 and *P* = 0.004, respectively). About three-quarters (73.4%, 102/139) of Unit staff had access to other in-country OH researchers; two-thirds (65.0%, 89/137) had internationally-based access. Most (87.7%, 121/138) Unit staff could publish findings in peer-reviewed national research journals; access increased to 90.1% (127/141) for international journals. Units undertaking OH research were more likely to have certain laboratory resources than those not, including for sample treatment (laboratory ovens (OR = 2.62, *P* = 0.040), microbiological incubators (OR = 2.53, *P* = 0.046), centrifuges (OR = 3.19, *P* = 0.011)), storage (refrigerators (OR = 2.80, *P* = 0.029), -20 °C (OR = 3.37, *P* = 0.008) and -80 °C (OR = 3.14, *P* = 0.023) freezers) and biosafety ((fume cupboards/biological safety cabinets (OR = 4.29, *P* = 0.003), disinfection and sterilisation of equipment (OR = 2.53, *P* = 0.046), laboratory able to handle BSL1 (OR = 3.18, *P* = 0.017), BSL2 (OR = 3.16, *P* = 0.030) or BSL3 (OR = 6.67, *P* = 0.044) samples)); there was borderline significance in relationships for certain other resources ((autoclave in laboratory (OR = 2.41, *P* = 0.062), effluent treatment (OR = 3.35, *P* = 0.054), and anteroom (OR = 5.86, *P* = 0.073)).

Respondents reported on topics (or disciplines) most studied by Unit researchers in the last five years in relation to the human, animal and environment dimensions, reporting on whether they considered research used OH approaches. There were patterns in whether approaches were reported as OH, and the human, animal and environment dimensions covered (Table 1); using a OH approach was more popular when the animal, human and environment, or animal and environment were examined, compared to when the animal and human, or human and environment were examined (Table 1 and Appendix A, Fig. 1). Patterns were statistically significant for some comparisons, dependent on topic frequency. For example, the most popular (top-half) topics examined using all triad dimensions were more than six times more likely to be reported as using OH approaches than the least popular (bottom-half) topics examined using the human and animal dimensions (Odds Ratio(OR) = 6.11, Lower Confidence Interval(LCI) = 1.23, Upper Confidence Interval(UCI) = 32.06, *P* > 0.030; this is illustrated using antimicrobial resistance versus biology in Fig. 4e and 4f, respectively). The most popular topics examined using all triad dimensions were also twenty-eight times more likely to be reported as using OH approaches compared to the least popular topics examined using the human and environment dimensions (OR = 28.54, LCI = 4.85, UCI = 204.78, *P* > 0.001; illustrated using antimicrobial resistance versus sanitation (Fig. 4e and 4g, respectively). The most popular topics examined using the human and animal dimensions were nearly five times more likely to be reported using OH approaches than the least popular topics examined using the human and environment dimensions, in a borderline relationship (OR = 4.67, LCI = 0.79, UCI = 32.50, *P* = 0.099); illustrated using antimicrobial resistance versus social sciences and humanities (Fig. 4e and 4h). Using OH approaches was also 19 times more likely to be reported for the most popular topics examined using the animal and environment dimensions compared to the least popular topics examined using the human and environment dimensions (OR = 18.98, LCI = 2.91, UCI = 148.79, *P* > 0.003; illustrated using antimicrobial resistance versus sanitation (Fig. 4e and 4g).

**Table 1:** The frequency that research on topics (or disciplines) was undertaken in Research Units in the last five years, reported by respondents in relation to whether the research examined the human, animal and environment dimensions. For some topics, Research Units more often used a One Health approach (more OH) compared to less often using One Health (Less OH) or an equal number reporting (Equal). As an example, for the topic AMR (antimicrobial resistance), for studies involving humans and animals (but not the environment), more Units reported using OH approaches (*n* = 8) than not using OH approaches (*n* = 4). Bold text depicts when the count of Units reporting working using One Health approaches was greater (than not using them), and ‘equal’ is when counts were the same. Numbers in brackets are counts.

	Dimensions examined for topic within research Unit (Counts for using OH versus not - NOH)			
Research topic	Animals & Humans	Humans & Environment	Animals & Environment	Animals, Humans & Environment
AMR	**Greater OH** (OH = 8, NOH = 4)	Less OH (OH = 1, NOH = 2)	**Greater OH** (OH = 2, NOH = 1)	**Greater OH** (OH = 8, NOH = 1)
Bacterial infections	Less OH (OH = 7, NOH = 8)	Less OH (OH = 0, NOH = 8)	Equal (OH = 1, NOH = 1)	**Greater OH** (OH = 7, NOH = 2)
Biology	Less OH (OH = 1, NOH = 3)	Less OH (OH = 0, NOH = 1)	Equal (OH2, NOH = 2)	Less OH (OH = 1, NOH = 3)
Biothreats	**Greater OH** (OH = 2, NOH = 1)	Equal (OH = 0, NOH = 0)	**Greater OH** (OH = 1, NOH = 0)	Less OH (OH = 1, NOH = 3)
Clinical medicine	Less OH (OH = 0, NOH = 4)	Less OH (OH = 0, NOH = 2)	**Greater OH** (OH = 1, NOH = 0)	Less OH (OH = 1, NOH = 3)
Earth and physical sciences	Equal (OH = 0, NOH = 0)	Less OH (OH = 0, NOH = 2)	Equal (OH = 1, NOH = 1)	Equal (OH = 1, NOH = 1)
Ecology	Equal (OH = 0, NOH = 0)	Less OH (OH = 0, NOH = 1)	Equal (OH = 2, NOH = 2)	**Greater OH** (OH = 5, NOH = 0)
Economics	Less OH (OH = 0, NOH = 2)	Less OH (OH = 0, NOH = 4)	Equal (OH = 0, NOH = 0)	Less OH (OH = 0, NOH = 2)
Ecosystem health	**Greater OH** (OH = 1, NOH = 0)	Less OH (OH = 0, NOH = 1)	Less OH (OH = 1 NOH = 2)	**Greater OH** (OH = 6, NOH = 3)
Engineering	Equal (OH = 0, NOH = 0)	Equal (OH = 0, NOH = 0)	**Greater OH** (OH = 1, NOH = 0)	Less OH (OH = 0, NOH = 1)
Environmental hazards exposure	Less OH (OH = 0, NOH = 2)	Less OH (OH = 0, NOH = 6)	Less OH (OH = 2, NOH = 3)	**Greater OH** (OH = 6, NOH = 5)
Food safety	Less OH (OH = 1, NOH = 3)	Less OH (OH = 0, NOH = 7)	**Greater OH** (OH = 2, NOH = 1)	**Greater OH** (OH = 12, NOH = 3)
Global health	Equal (OH = 1, NOH = 1)	Less OH (OH = 1, NOH = 2)	Equal (OH = 0, NOH = 0)	**Greater OH** (OH = 6, NOH = 1)
Health sciences	Less OH (OH = 1, NOH = 3)	Less OH (OH = 0, NOH = 3)	**Greater OH** (OH = 1, NOH-0)	**Greater OH** (OH = 6, NOH = 2)
Human-animal bond	Equal (OH = 5, NOH = 5)	Equal (OH = 0, NOH = 0)	Equal (OH = 0, NOH = 0)	**Greater OH** (OH = 3, NOH = 2)
Metabolic disorders	Less OH (OH = 0, NOH = 2)	Less OH (OH = 0, NOH = 1)	Equal (OH = 0, NOH = 0)	**Greater OH** (OH = 2, NOH = 0)
Molecular and microbiology	Equal (OH = 4, NOH = 4)	Equal (OH = 1, NOH = 1)	Equal (OH = 1, NOH = 1)	**Greater OH** (OH = 9, NOH = 1)
Parasite infections	Less OH (OH = 2, NOH = 9)	Less OH (OH = 1, NOH = 4)	Equal (OH = 1, NOH = 1)	**Greater OH** (OH = 7, NOH = 1)
Population health	Less OH (OH = 1, NOH = 2)	Equal (OH = 3, NOH = 3)	Equal (OH = 0, NOH = 0)	**Greater OH** (OH = 4, NOH = 0)
Public health	Equal (OH = 3, NOH = 3)	Less OH (OH = 0, NOH = 4)	Less OH (OH = 0, NOH = 1)	**Greater OH** (OH = 15, NOH = 0)
Sanitation	Equal (OH = 2, NOH = 2)	Less OH (OH = 1, NOH = 5)	Equal (OH = 1, NOH = 1)	**Greater OH** (OH = 7, NOH = 2)
Social sciences and humanities	**Greater OH** (OH = 3, NOH = 0)	Less OH (OH = 1, NOH = 4)	Equal (OH = 0, NOH = 0)	**Greater OH** (OH = 2, NOH = 1)
Surveillance	**Greater OH** (OH = 3, NOH = 0)	Less OH (OH = 0, NOH = 1)	Equal (OH = 0, NOH = 0)	**Greater OH** (OH = 5, NOH = 3)
Vaccines and therapeutics	Less OH (OH = 4, NOH = 5)	Equal (OH = 0, NOH = 0)	**Greater OH** (OH = 1, NOH = 0)	Equal (OH = 1, NOH = 1)
Vectorborne infections	Equal (OH = 4, NOH = 4)	Equal (OH = 2, NOH = 2)	**Greater OH** (OH = 2, NOH = 1)	**Greater OH** (OH = 8, NOH = 4)
Vector control	Less OH (OH = 1, NOH = 3)	Less OH (OH = 2, NOH = 3)	Equal (OH = 2, NOH = 2)	**Greater OH** (OH = 6, NOH = 5)

## 4 Discussion

Following calls to urgently identify and fill gaps in countries' health security systems and promote OH capacity building [11,12], this survey aimed to better understand Horn of Africa OH research capabilities. A key finding was that while four-fifths of Units acknowledged some OH research, there was bias towards human-focused OH triad dimensions including at the human/animal/environment-interface and human/animal-interface. This reflects previous patterns from bibliometric analyses [13,14] and OH networks, where 78% used OH approaches, but 16% did not identify themselves with OH-aligned terms, and a third did not report activities related to the external environment and its effects on human or animal health, list the environment/ecosystem as an area of concern or network-focus, or include appropriate authors in research [5]. This bias is also reflected in OH approaches being more often reported when all dimensions or the animal/environment dimensions were examined compared to the human and either animal or environment dimensions. The approach we used, utilising ternary diagrams, acts as an evaluation of understanding for the topics and disciplines in which OH approaches can be used; ideally, research on any topic should consider whether OH approaches are needed. A lack of integration across the OH triad is discussed in relation to improving communication as a OH competency [15], and illustrated by neglect of the environment in 31% of OH networks [5] and when described in an AMR in the environment case study [16]. It was also previously reported when government-funded centres for emerging infectious diseases were hosted by medical schools working with similar Units, limiting wildlife and veterinary scientist links, and removing social, economist, and environmental health scientist engagement [17]. The medical science cluster also had the most connections to other clusters in bibliometric analysis [14]. Differences in research focus within interpretation of One Heath by survey respondents also reflect neglect of the environment in the OH triad [14,16,[18], [19], [20]], a lack of standardised education on OH and difficulties in determining a OH definition [21] leading to improvements [22]. Our study provides new insights on education; we detected subject bias in Unit staff impacting environment-focus in research, because higher environmental science degrees were only more likely with greater research focus in Units compared to clinical medical or veterinary degrees, or higher basic/applied science degrees. The need for focused OH approaches when studying certain topics reflects links between disciplines from previous analysis of the “OH cosmos” [21] e.g., zoonoses (encompassing bacterial, parasite and vectorborne infection topics) with food safety, and education with epidemiology, human medicine and veterinary medicine (encompassing AMR, global health, health sciences and public health topics), ecology, and molecular/microbiology.

Two-thirds of Units we examined receive external research funding; encouraging given a reported lack of effective OH research funding mechanisms [18]. Our finding that RIs and other organisations were more likely to receive funding than universities, contrasts with (differently-focused) studies of OH capacity building programmes in south/south-east Asia with 80% and 56% of these based in universities [23], and OH networks, where 76% involved academics and 78% involved government bodies [5,20]. There was also a lack of buy-in from (not fully-defined) ‘local’ research and funding agencies (including universities) in capacity building programmes [23] in juxtaposition to our work, which suggests good national buy-in (inferring ownership) of research, with international government organisations being second largest donors. It also reflects our Units' focus, with the greatest proportion being public-sector, focused on research and providing HE (including human and veterinary training).

Proportions of Units undertaking OH research within different sectors were similar in this study to previous patterns [6], and our results also highlight under-representation of NGO and private-sector stakeholders in OH research funding. Additional to funding some research, NGOs receive international biomedical research and government funding, and are provided with research reports from Units, suggesting they have greater focus on delivering and consuming research than funding work. Differing opinions on whether the private sector should be involved in OH research have been discussed, with experts arguing that this should be on a case-by-case basis or by increasing involvement of civil society [20]. The public-focus of OH research was also reflected in many Units engaging with and most providing reports to national government policy-makers, followed by regional, then local; the under-representation of local community organisations has previously been identified [5,20]. It suggests integration lacking across government hierarchies during policy-development, purportedly due to absent social science insights to facilitate implementation and management, and international organisations dominating policy-clusters [14]. Additionally, results utilisation from national reports is impacted by research article authors preferences to quote peer-reviewed articles rather than reports [13,14]. International sphere dominance is further illustrated in researcher's greater access to international compared to national peer-reviewed research journals, and by their relatively high access to other international compared to within-country OH researchers (a pattern also reflected in national compared to international laboratory links [6]). The importance of engagement with non-academic groups to develop diverse approaches and solutions is highlighted because research collaborations, even for OH, are likely to be impacted by physical and academic closeness [14].

Four-fifths of surveyed Units had laboratory facilities compared to two-thirds previously [6], though the surveys covered different geographical regions (Horn region versus 42 Africa countries, respectively) and had different foci (facilities for human and/or animal research versus (human) health facilities, respectively). Interestingly, despite this, the proportion of Units able to process either human or animal samples was identical (at two-thirds), with over half processing both. This may reflect the high proportion of Units containing National Reference Laboratories (four-fifths, compared to 45% previously [6]), perhaps reflecting well-resourced respondents. Resource availability is further reflected in laboratory equipment and biosafety facilities availability, which mirror (with low proportions overall) supplies in institutions handling high risk infectious agents and RIs generally, respectively [6]. Our survey provides evidence that either OH approaches are more likely used if laboratory biosafety resources, sample treatment and storage facilities are available, or that they encourage such facilities' development. The same is not true for financial laboratory resources, however; we identified no difference compared to research without OH approaches, in addition to the proportion of Units reporting having funding for equipment purchase, supplies and maintenance, and funding for staff training on technical and safety issues, being nearly half that previously identified [6]. This issue may either be worse or have worsened for the Horn region compared to wider Africa.

This work was undertaken as a part of the HORN project; reflected in reported memberships of OH networks. During study recruitment, steps were taken to minimise biases in respondents; invited Key Informants may have had prior contract with HORN but they could also have been invited by collaborators or have been identified from the internet or have been authors on Horn-region research outputs. The 159 responses dwarf 11 main HORN partner organisations, suggesting recruitment success. As this study was delivered online, we acknowledge that analysed Units may be biased by internet connectivity, however the survey could be completed using a Smartphone and in multiple stages, minimising this impact.

## 5 Conclusions

The apparent neglect of the environment in the OH triad which this research highlights, reflects previous biases towards certain OH research activities, e.g., surveillance and monitoring, and development of new products, compared to implementation work in low/middle-income settings or policy development [5] in which the environment might need more consideration. Spikes in topic interests cause suboptimal strategic planning, coordination, and stakeholder engagement, marginalising more complex drivers of disease such as ecosystem change and socio-political dynamics [5,24]; there are persistent silos in the OH triad dimensions [14] meaning that vital strengthening of intersectoral collaboration and knowledge-sharing is necessary to aid implementation and management of policy within all levels of decision-makers [13]. Our work suggests that in the Horn region, collaboration between academia and government happens but does not always translate through government hierarchies during policy-development, and it may be driven by international organisations as opposed to local, despite national-level buy-in. Whilst we highlight a bias towards international research collaborations, the Units investigated produce national reports. These results should be fully utilised in future research planning, and collaborative platforms and institutions must focus on engaging with civil society, generating political support across government hierarchies [20] and aiding adaptation of interventions to local contexts [5]. This will facilitate OH advocacy from research through to policy implementation. Our research also highlights reasons behind environment dimension neglect; Units omit employing a balance of researchers skilled in each of the three triad dimensions, and continue with human-centric, funding-seeking motivations [5]. They may also not consider using OH approaches to research every topic, or within every discipline. Environmental perspectives and solutions take longer to understand and investigate; future work must better account for and consider interventions to ameliorate environment impacts, creating more balanced, strengthened global health outcomes. Finally, while we highlight good laboratory resource availability for aspects of OH research, Units are still lacking regular funding for laboratory equipment purchase, supplies and maintenance, for staff training on technical and safety issues; this may have worsened compared to the earlier sub-Saharan Africa [6] study, suggesting urgent improvements are needed in short-term research planning.

## Contributors

KMM and MB conceptualised and designed the research, KMM and MC undertook acquisition of the data, KMM conducted analyses and interpretation, and drafted the manuscript. MC and MB provided inputs for revisions of the manuscript. All authors approved the final version.

## Ethical approval and informed consent

The study was carried out in accordance with The Code of Ethics of the World Medical Association (Declaration of Helsinki) for experiments involving humans (Version 2008). The research protocol was approved by University of Liverpool Veterinary Research Ethics Committees (Ref. VREC930), with survey data stored in UK. All study participants provided informed consent (see Appendix A for full details).

## Availability of materials

Following appropriate ethical permissions, the intermediate and final datasets are available on reasonable request to the corresponding author.

## Role of the funding source

We thank the funders of this research for their support (10.13039/501100000268BBSRC project number BB/P027954/1) and confirm that the study funders had no role in study design, in the collection, analysis and interpretation of data, in the writing of the report, and in the decision to submit the article for publication.

## Declaration of Competing Interest

We declare no competing interests.

## Data Availability

Data will be made available on request.

## Appendix A Supplementary data

Appendix A including ethical approval details, recruitment information, further results, and the extended version of the main articles' Fig. 4e-h (Appendix A, Figure 1) Image 1
