# Supplementary material for: Capacity for One Health research in the Horn of Africa
Source: One Health. 2023 Apr 28;16:100549. doi: 10.1016/j.onehlt.2023.100549 (PMC10288085; doi:10.1016/j.onehlt.2023.100549)
Supplement: Supplementary file 1 — Appendix A including ethical approval details, recruitment information, further results, and the extended version of the main articles' Fig. 4e-h (Appendix A, Figure 1) [file mmc1.pdf]

Appendix - Capacity for One Health research in the Horn of Africa

Contents

Ethical Approval 1

Recruitment information 1

Further results 1

Figure 1. The number of One Health dimensions (described by symbol size, including human, animal, and environment) examined for research topics/disciplines within Research Units (in the last five years). 2

References 5

Ethical Approval

The study was carried out in accordance with The Code of Ethics of the World Medical Association (Declaration of Helsinki) for experiments involving humans (Version 2008). The research protocol was approved by University of Liverpool Veterinary Research Ethics Committees (Ref. VREC930), with survey data stored in UK.

Recruitment information

Potential Key Informants were sent a specific Survey Invitation e-mail asking if they were willing to fill in the questionnaire. Upon a positive response, a link unique to the survey (administered using JISC Online Surveys[1]) was then forwarded to them, including a survey Information Sheet, Consent Form, and main survey questions. If the respondent felt they were not the most suitable person to respond, they were asked to recommend an alternative Key Informant, (or they could forward the e-mail themselves, to the most suitable recipient). A single reminder e-mail was sent to potential participants two weeks after the initial invitation. As a continuous internet connection to complete the survey might not always be available, a downloadable version could also be e-mailed back to an anonymised address; such survey responses would be entered into the online survey facility ensuring that Personally Identifiable Information (PII) was treated in the same anonymous manner for all respondents. The survey questions and responses were written in English, and none were compulsory. Participants were asked to provide informed consent to take part in the survey. They were informed that their participation was voluntary, they were free to withdraw their responses at any time, and their data was confidential. Participation was encouraged by participants being eligible for entry into a draw to win books for their research Unit. Survey respondents were asked to confirm their main role in their Unit, and highest qualification; PII was only used to confirm individuals taking part. If research was not undertaken in units, staff were asked basic questions on research resources availability.

Further results

There were no funding differences as a result of units' research sector e.g., public/government funded, private for-profit or not-for-profit, NGO (P=0·414) or primary function e.g. conducting research, higher education, pharmaceutical, civil service/government analysis, human/veterinary health services or training (P=0·831). No factors influenced unit researchers' qualifications other than those in the main results (P>0·050). Whether Units had local, regional, national or international-focus, their country or official/working language, the proportions of national staff or women employed, whether they were able to process human laboratory samples, whether they hosted a National Reference Laboratory, or whether they were able to send samples to other laboratories within-country, in neighbouring countries or globally were not related to One Health working (P>0·05).

Figure 1. Patterns in whether research approaches for topics (or disciplines) were more often reported as One Health, and whether the research examined the human, animal and environment dimensions. The counts describe the frequency of Units reporting undertaking research for each topic (or discipline) and the dimensions their research covered. One Health approaches (black diamonds) were more often reported (compared to not using One Health approaches – yellow circles, i.e., black diamonds larger than yellow circles) when the animal, human and environment (central), or animal and environment (centre of right axis) were examined, compared to when the animal and human (centre of left axis), or human and environment (centre of bottom axis) were examined. A point but no diamond or circle depicts a count of zero, and a formal statistical comparison is presented in the main study results.

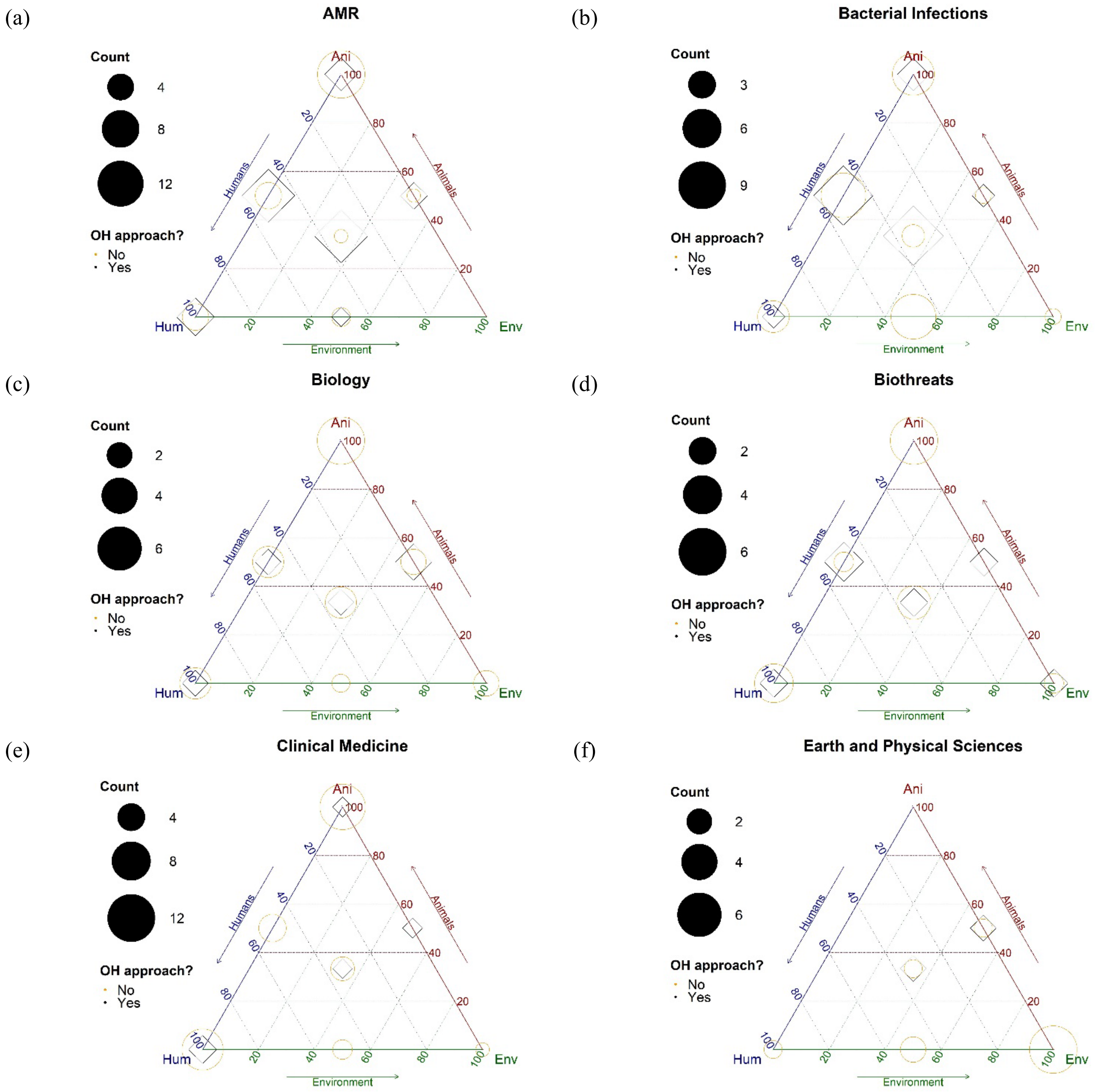

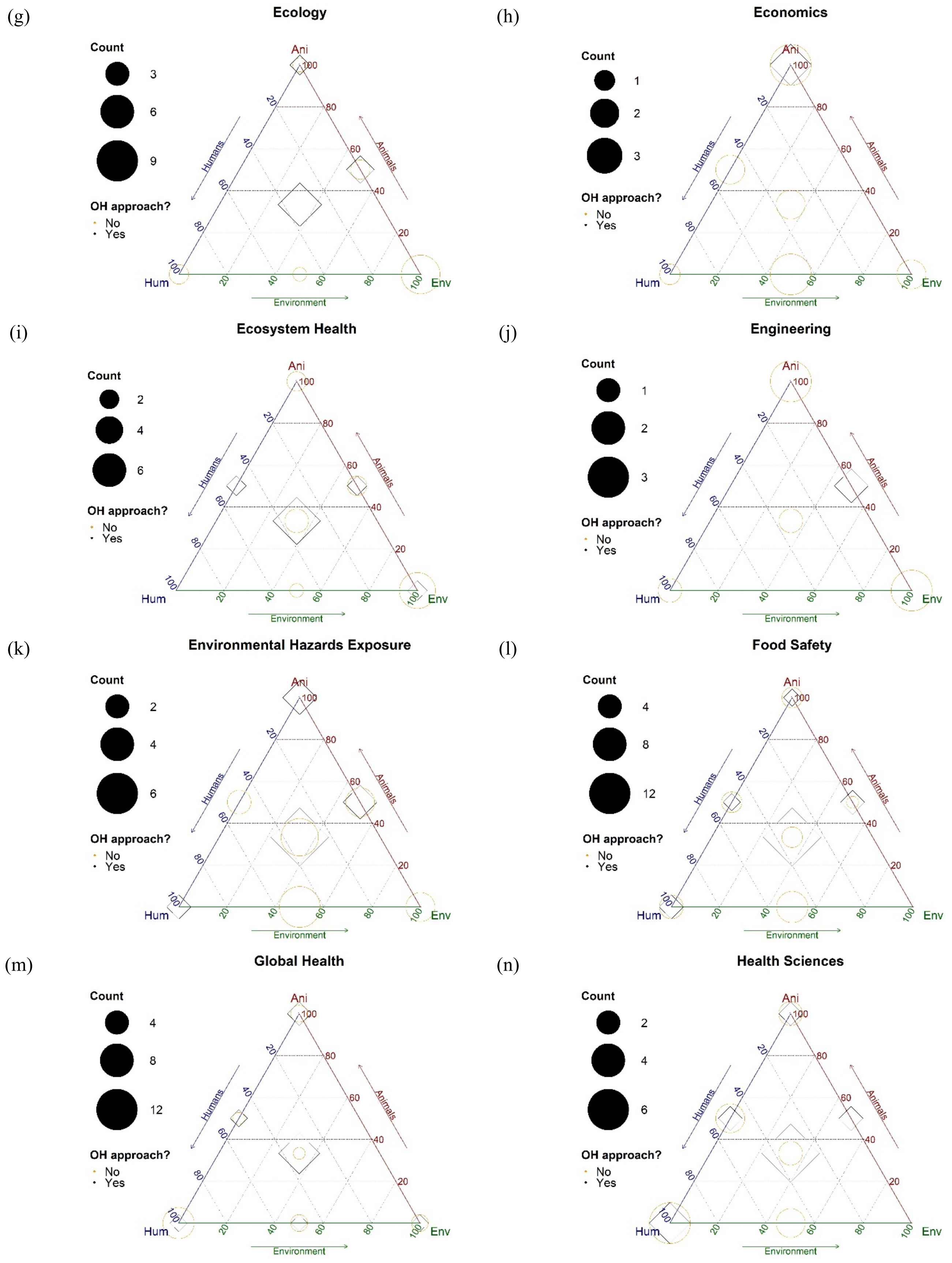
(g)
Ecology
Count
3
6
9
OH approach?
No
Yes
(h)
Economics
Count
1
2
3
OH approach?
No
Yes
(i)
Ecosystem Health
Count
2
4
6
OH approach?
No
Yes
(j)
Engineering
Count
1
2
3
OH approach?
No
Yes
(k)
Environmental Hazards Exposure
Count
2
4
6
OH approach?
No
Yes
(l)
Food Safety
Count
4
8
12
OH approach?
No
Yes
(m)
Global Health
Count
4
8
12
OH approach?
No
Yes
(n)
Health Sciences
Count
2
4
6
OH approach?
No
Yes
Ani
Hum
Env
Humans
Animals
Environment

(o) Human-Animal Bond

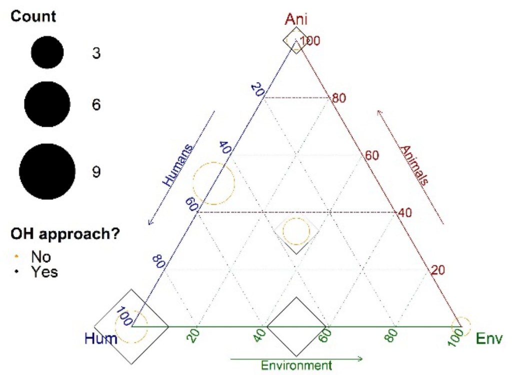

(p) Metabolic Disorders

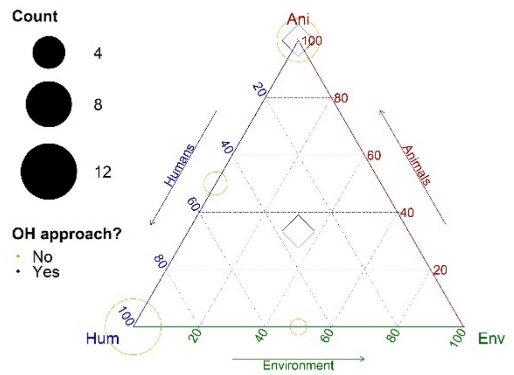

(q) Molecular Microbiology

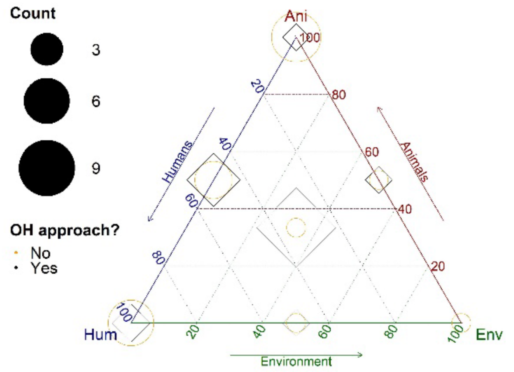

(r) Parasite Infections

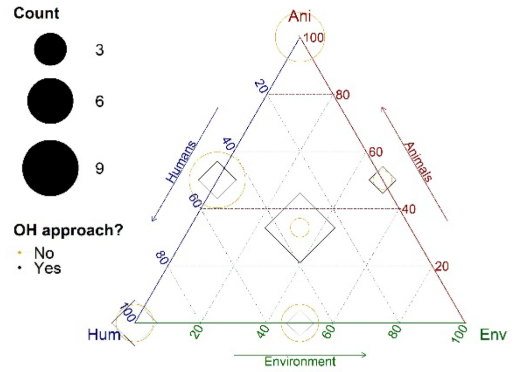

(s) Population Health

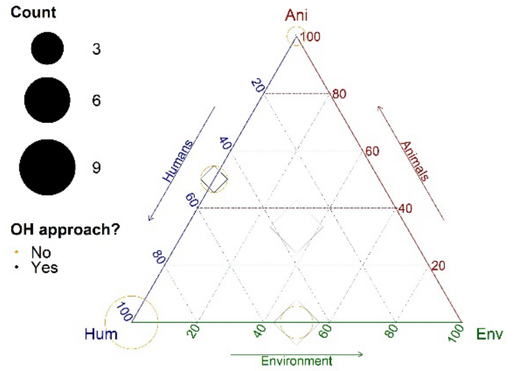

(t) Public Health

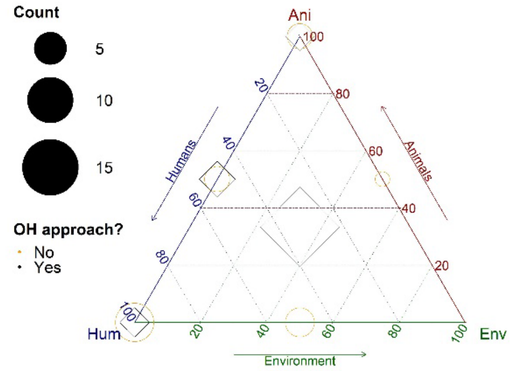

(u) Sanitation

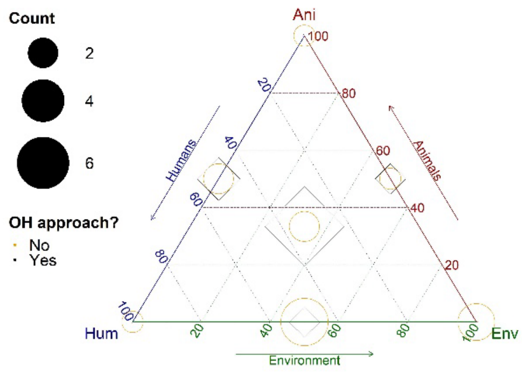

(v) Social Science & Humanities

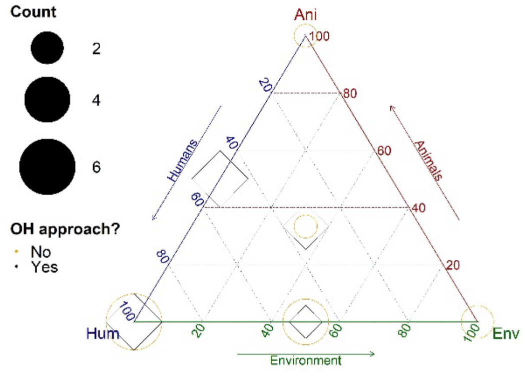

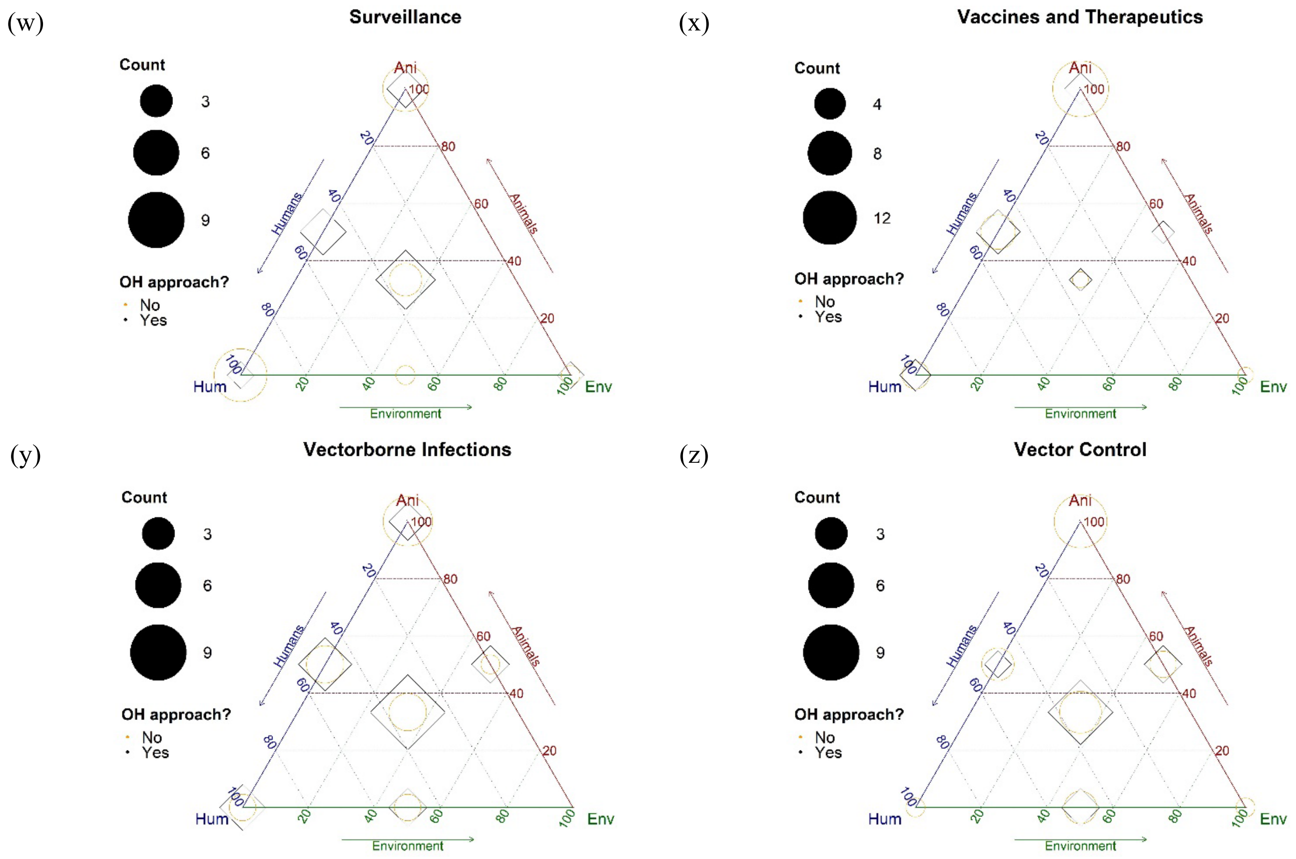

References

1. Anonymous. JISC Online Surveys. 2021. https://www.onlinesurveys.ac.uk/
